# Crosslinking Efficacy and Cytotoxicity of Genipin and Its Activated Form Prepared by Warming It in a Phosphate Buffer: A Comparative Study

**DOI:** 10.3390/ma14216600

**Published:** 2021-11-02

**Authors:** Takeya Kawamura, Shunji Yunoki, Yoshimi Ohyabu, Toshio Uraoka, Kazuaki Muramatsu

**Affiliations:** 1 Biotechnology Group, Tokyo Metropolitan Industrial Technology Research Institute (TIRI), 2-4-10 Aomi, Koto-ku, Tokyo 135-0064, Japan; 19rmb08@ms.dendai.ac.jp (T.K.); yoshimi.ohyabu@iri-tokyo.jp (Y.O.); 2 School of Science and Engineering, Tokyo Denki University, Ishizaka, Hatoyama-cho, Hiki-gun 350-0394, Japan; k-muramatsu@mail.dendai.ac.jp; 3 Department of Gastroenterology and Hepatology, Gunma University School of Medicine, 3-39-22 Showa-machi, Maebashi 371-8514, Japan; uraoka@gunma-u.ac.jp

**Keywords:** genipin, crosslinking, hydrogel, aldehyde, cytotoxicity, IC50, injectable

## Abstract

The aim of the present study was to compare the acute and cumulative cytotoxicity of intact (n-GE) and warmed genipin (w-GE), while investigating the differences in crosslinking capabilities of these two genipins by rheological and mechanical tests. The n-GE solution was prepared by dissolving genipin powder in a sodium phosphate buffer solution. The w-GE solution was prepared by warming the n-GE solution at 37 °C for 24 h. The mechanical tests for chitosan (CH)/genipin gels showed the crosslinking rate of w-GE was much greater than that of n-GE up until 6 h after preparation, whereas the degree of crosslinking of CH/n-GE gels became higher at 12 h. The ISO 10993-5 standard method, which is established specifically for evaluating cumulative cytotoxicity, determined equivalent IC50 for w-GE (0.173 mM) and n-GE (0.166 mM). On the other hand, custom-made cytotoxicity tests using a WST-8 assay after 1 h of cultivation showed that the acute cytotoxicity of w-GE was significantly higher than that of n-GE at concentrations between 0.1–5 mM. The acute cytotoxicity of w-GE should be taken into consideration in its practical uses, despite the fact that the much faster crosslinking of w-GE is useful as an effective cross linker for in-situ forming gels.

## 1. Introduction

The mechanical properties and biological stabilities of biopolymers (proteins and polysaccharides) are generally lower than those of synthetic polymers, such as polyesters. Therefore, the introduction of chemical crosslinking is a simple and effective technique to improve said properties. Various chemical cross linkers have been used for crosslinking biopolymers in tissue fixation [1]. Glutaraldehyde (GA) and formaldehyde have been clinically used because of their excellent crosslinking efficacies. However, the toxicity of chemical cross linkers sometimes causes clinical drawbacks [2], suggesting a clear tradeoff between crosslinking efficacies and toxicity.

Genipin is a natural cross linker of proteins extracted from gardenia fruit and has been extensively used as a low cytotoxicity cross linker for biomaterials, including biological tissues [3,4], hydrogel bodies [5,6,7,8], in-situ-forming hydrogels [9], micro-particles [10], and porous scaffolds of biopolymers [11]. Some of the genipin-crosslinked materials are demonstrated to be effective for biomedical applications, such as a scaffold for tissue engineering in intervertebral disk [11], articular cartilage [5], and as a drug delivery carrier for treating spinal cord injury [12]. The genipin molecule reacts to a pair of free amine residues on proteins or polysaccharides [13], resulting in crosslinking between them, in which ring-opening polymerization of genipin could be included [14,15]. This reaction between a pair of free amine residues is similar to that of GA; one of the most conventional and effective protein cross linkers.

The advantage of genipin over GA is in its biological safety while exhibiting comparable crosslinking efficacies. Tensile stress and biological stability of biomaterials (reflecting crosslinking efficacies) are comparable between genipin and GA, despite in vivo inflammatory reactions to genipin being much lower [16]. In addition, it has been reported that cells can tolerate in culture media containing genipin at concentrations below 0.5–1.0 mM [9,17,18,19].

A shortcoming of genipin is its slow reaction rate to biopolymers when compared to GA, specifically at ambient temperatures [20]. However, we found that this limitation could be overcome by a simple warming of genipin in a sodium phosphate buffer solution [21]. A neutral collagen solution containing warmed genipin exhibited gelation much faster than a solution containing intact genipin. This characteristic is especially beneficial for submucosal injectable materials in endoscopic resection [22]. On the other hand, the enhancement of its cytotoxicity due to the warming process is of concern. We speculated that the cytotoxicity of warmed and intact genipins are different because the former genipin has aldehyde groups [21].

The aim of the present study was to compare the cytotoxicity of warmed and intact genipins while investigating the differences in their crosslinking efficacies by rheological and mechanical tests. The acute and cumulative cytotoxicity of these genipins were evaluated by short-term and long-term cell culture tests, respectively. The crosslinking efficacies (i.e., reaction rates and degrees of crosslinking) were evaluated by measuring the gelation of chitosan (CH) solutions containing genipins. Our data could not only cultivate better understanding of this useful crosslinker but also contribute to various biomaterial designs.

## 2. Materials and Methods

### 2.1. Materials

Chitosan (200–600 mPa·s; 0.5% in 0.5% acetic acid at 20 °C, Tokyo Kasei Kogyo, Tokyo, Japan), genipin (FUJIFILM Wako Pure Chemical Corporation, Osaka, Japan), acetic acid (FUJIFILM Wako Pure Chemical Corporation, Japan), disodium hydrogen phosphate (Na_2_HPO_4_; FUJIFILM Wako Pure Chemical Corporation, Japan), sodium dihydrogen phosphate (NaH_2_PO_4_; FUJIFILM Wako Pure Chemical Corporation, Japan), Giemsa Stain Solution (Merck, Darmstadt, Germany), 10% formalin neutral buffer solution (FUJIFILM Wako Pure Chemical Corporation, Japan), Dulbecco’s PBS (PBS; Sigma-Aldrich, St. Louis, MO, USA), polyetherurethane (PU) film containing 0.1% zinc diethyldithiocarbamate (ZDEC) (ZDEC-PU; Hatano Research Institute, Hadano, Japan), PU film containing 0.25% zinc dibuthyldithiocarbamate (ZDBC) (ZDBC-PU; Hatano Research Institute, Hadano, Japan), high-density polyethylene sheet (HDPE; Hatano Research Institute, Hadano, Japan), Chinese hamster lung fibroblast V79 cells (V79 cells; RIKEN RCB0008 and Japan Health Sciences Foundation JCRB0603), Eagle’s minimum essential medium (MEM; Sigma-Aldrich, St. Louis, MO, USA), fetal bovine serum (FBS; Corning Cellgro, Manassas, VA, USA), 0.5% and 0.25% trypsin solution (FUJIFILM Wako Pure Chemical Corporation, Japan and Thermo Scientific, Carlsbad, CA, USA, respectively), and CELL COUNTING KIT-8 (Dojindo Laboratories, Kumamoto, Japan) were used in this study.

### 2.2. Preparation of Test Solutions

#### 2.2.1. Preparation of CH Solutions

CH powder was dissolved in a 0.5 M acetic acid solution at a concentration of 1% using an agitation apparatus with an impeller. The viscous solution was dialyzed against pure water using a dialysis tube composed of regenerated cellulose (Visking tube; MWCO = 12–14 kDa, Japan Medical Science, Osaka, Japan), resulting in dilution of CH by osmotic pressure. The concentration of CH in the dialyzed solutions was determined from its dry weight. The CH solution was concentrated with a rotary evaporator at 40 °C to achieve concentrations of 0.5% or 1%.

#### 2.2.2. Preparation of Sodium Phosphate Buffers

Sodium phosphate buffers (designated “PB-X”) were prepared by mixing X mM of Na_2_HPO_4_ and X mM of NaH_2_PO_4_ solutions. The pH was adjusted to 7 by adding varying volume ratios of the two sodium phosphate solutions.

#### 2.2.3. Preparation of Genipin Solution

Genipin powder was dissolved in PB-20 for rheological and mechanical tests and in PB-50 for cell culture tests at concentrations between 2–50 mM. If the solutions were immediately used after their preparation, the genipin was designated “n-GE”. On the other hand, n-GE solution which was warmed at 37 °C for 24 h to activate genipin was designated “w-GE” [21]. The w-GE solution was used after cooling at ambient temperature.

#### 2.2.4. Preparation of CH/Genipin Solutions

The 0.5% CH solution was mixed with an equal volume of w-GE or n-GE solution to obtain 0.25% CH/1–5 mM genipin solutions. The 1% CH solution was mixed with 5 mM w-GE or n-GE solution at a volume ratio of 4:1 to obtain 0.8% CH/1 mM genipin solutions. The two series of CH/genipin solutions were subjected to the following rheological and mechanical tests.

### 2.3. Infrared Analyses of Genipins and Crosslinked CH

Fourier transform infrared (FTIR) spectra of w-GE and n-GE were recorded on a Bruker ALPHA II FTIR spectrometer (Bruker Optics, Billerica, MA, USA) equipped with a Platinum ATR. The w-GE and n-GE solutions were freeze-dried with a vacuum freeze drier (FDU-2110, EYELA, Tokyo, Japan) to obtain dried specimens. The spectra were recorded in the 4000 to 400 cm^−1^ range with a resolution of 2 cm^−1^.

### 2.4. Rheological Tests of CH/Genipin Solutions

The gelation of CH/genipin solutions was monitored using a rotational rheometer equipped with a Peltier temperature controller (HAAKE MARS III, Thermo Fisher Scientific, Waltham, MA, USA) in accordance with our previously described method [23] to evaluate the crosslinking efficacies of genipin. Briefly, approximately 4 mL aliquots of the 0.25% CH/1–5 mM genipin solutions were poured onto the bottom plate of a parallel-plate sensor (diameter 60 mm), maintained at 37 °C, and dynamic viscoelastic measurements (oscillation frequency, 1 Hz; shear stress, 1 Pa) were initiated. Changes in storage modulus (G′) were registered throughout the tests (*n* = 3).

### 2.5. Mechanical Tests of CH/Genipin Solutions

Elastic moduli of the CH/genipin mixed gels were evaluated by penetration tests using a mechanical tester (TA.XTplus; Stable Micro Systems, Godalming, UK) according to our previously described method [21], with slight modifications. Aliquots of 3 g of the 0.8% CH/1 mM genipin solutions were poured into polystyrene biological dishes (diameter 35 mm) and placed on the surface of a water bath at a temperature of 37 °C to warm them up rapidly. In another set of conditions, an aliquot of 24 g of the CH/n-GE solution was poured into a Φ 100 mm dish, where it was subjected to the test for the shortest warming period (4 h). After 30 min of warming, the dishes with lids were sealed with a paraffin film to avoid drying of the gels and moved to an incubator at a temperature of 37 °C. After the pre-determined periods of incubation (≤7 days), the dishes containing gels were subjected to penetration tests. The dishes were put on aluminum disks (diameter 32 mm, thickness 3 mm) to prevent deformations of the bottoms of the plates during testing. The centers of the gels (*n* = 5) were probed with a cylindrical stainless probe (10 mm in diameter for Φ 35 mm dishes; 20 mm in diameter for Φ 100 mm dish) at a cross-head speed of 0.2 mm/s and stress–strain curves were obtained. The elastic modulus was calculated from the slope of the stress–strain curve in its linear region (strain from 0.01 to 0.05), wherein consolidation of the gels by the biological plates did not affect the mechanical data.

### 2.6. Acute Cytotoxicity Tests of Genipin

The acute cytotoxicity of w-GE and n-GE were evaluated by a custom-made method using a conventional WST-8 assay. Cell culture was carried out in a CO_2_ incubator (CO_2_ concentration = 5%) at 37 °C. Briefly, serum-free media containing various concentrations of w-GE and n-GE (0.02–5 mM) were prepared by the addition of 50 mM genipin solutions in PB-50 to MEM. V79 cells proliferated using MEM containing 10% FBS were collected and seeded onto 48-well biological plates at a density of 1 × 10^4^ cells/well. After a 6-h incubation to allow for cell attachment, the medium was replaced with serum-free medium containing genipins. After 1-h of incubation, each well was washed twice with PBS and then subjected to a WST-8 assay using CCK-8. The live cells were stained with formazan and measured by ABS at 450 nm. Percent cell viability was calculated as the ratio of each ABS (genipin concentrations 0.02–5 mM) to that of the control (n-GE concentration = 0 mM).

### 2.7. Cumulative Cytotoxicity Tests of Genipin

The cumulative cytotoxicity of w-GE and n-GE were evaluated by the International Organization for Standardization (ISO) 10993-5 standard test method (Biological evaluation of medical devices―Tests for in vitro cytotoxicity), in which genipins were added directly to cell culture media instead of adding extracts from genipin-containing medical devices. Briefly, V79 cells were first cultured using MEM containing 10% FBS in T75 flasks to obtain sufficient cell numbers. They were subsequently seeded onto 24-well biological plates at a concentration of 50 colonies per well. After 20 h of incubation, the medium was replaced with medium containing various concentrations of w-GE and n-GE (0–0.5 mM). These mixtures were prepared immediately before use in order to minimize reactions of genipins with amino acids and proteins in the medium. After 7 days of incubation, colonies were fixed with 10% formalin neutral buffer and then stained with Giemsa. The colonies were counted manually using a stereo microscope. Percent colony formations were determined by comparing the numbers of colonies observed in genipin-containing media to those in media containing no genipin. The 50% inhibitory concentrations (IC50) were determined by the following equations [24]:

IC50 = 10^y^, (1)

y = Log(A/B)·(50 − C)/(D − C) + Log(B),(2)

where A and B are, respectively, the higher and lower genipin concentrations closest to 50%. C and D are percent inhibitory at A and B, respectively.

The validation of the cumulative cytotoxicity tests was then carried out. Parts of ZDEC-PU and ZDBC-PU were immersed in MEM containing 10% FBS at a concentration of 1 g/10 mL, and subsequently incubated at 37 °C for 24 h. The extracts obtained were then put through the above cytotoxicity tests in the same manner as that for genipin-containing MEM. When the IC50 of the extracts from ZDEC-PU and ZDBC-PU were <7% and <80%, respectively, and there was no significant difference between the colony numbers between the extracts from HDPE and the control, the cytotoxicity tests were said to be successfully validated.

### 2.8. Statistics

Cytotoxicity data of each genipin concentration were compared using a one-way analysis of variance (ANOVA). Significant differences between groups were identified using Tukey’s test and were considered significant when *p* < 0.05. Cytotoxicity data of w-GE and n-GE concentrations were compared using Student’s t-test to identify statistical significance (*p* < 0.05).

## 3. Results

### 3.1. Short-Term Gelation Monitoring of CH/Genipin Solutions

**Figure 1:**
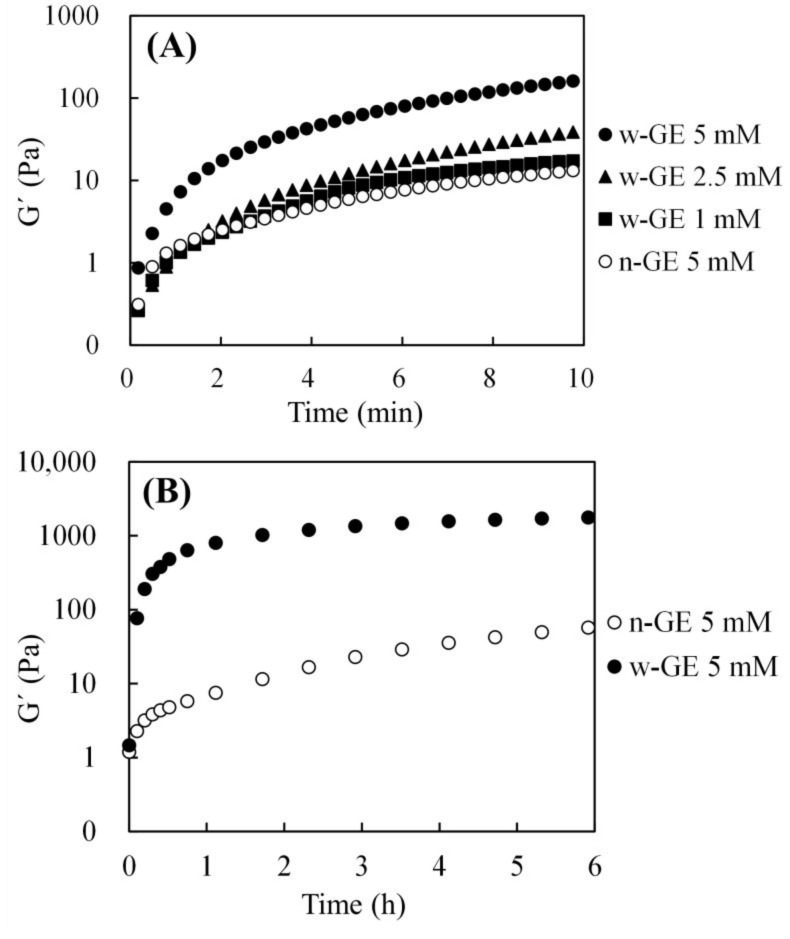
Representative time-dependent changes of shear storage modulus (G′) for 0.25% CH solutions containing 1–5 mM of w-GE, as well as 5 mM of intact genipin (n-GE). G′ was monitored for 10 min (**A**) and 6 h (**B**).

Figure 1 shows the representative gelation curves for the 0.25% CH/genipin solutions obtained by the dynamic viscoelastic measurements with the rheometer at 37 °C, exhibiting the differences of immediate crosslinking rates between w-GE and n-GE. The sharpest increases in G′ was observed for the solution containing 5 mM of w-GE immediately after the measurement initiated (i.e., immediately after CH and w-GE solutions were mixed) (Figure 1A). In contrast, the solution containing 5 mM of n-GE exhibited the slowest increase in G′, which was comparable to that of 1 mM of w-GE. The fastest gelation rate was observed in w-GE (5 mM), followed by w-GE (2.5 mM), and w-GE (1 mM), which was roughly equivalent to n-GE (5 mM).

The monitoring was continued for 6 h to compare time-dependent changes in crosslinking rates of w-GE and n-GE at the same concentration for a longer period (Figure 1B). The logarithmic increase in G′ observed in the CH/w-GE solution ended within 1 h, and then the increase became slower, resulting in a G′ of 1996 ± 52 Pa at 6 h. In contrast, the CH/n-GE solution did not exhibit a logarithmic increase in G′ at the early stages of the measurement and moved to a linear increase in G′ after 2 h, resulting in a G′ of 89 ± 4 Pa at 6 h.

**Figure 2:**
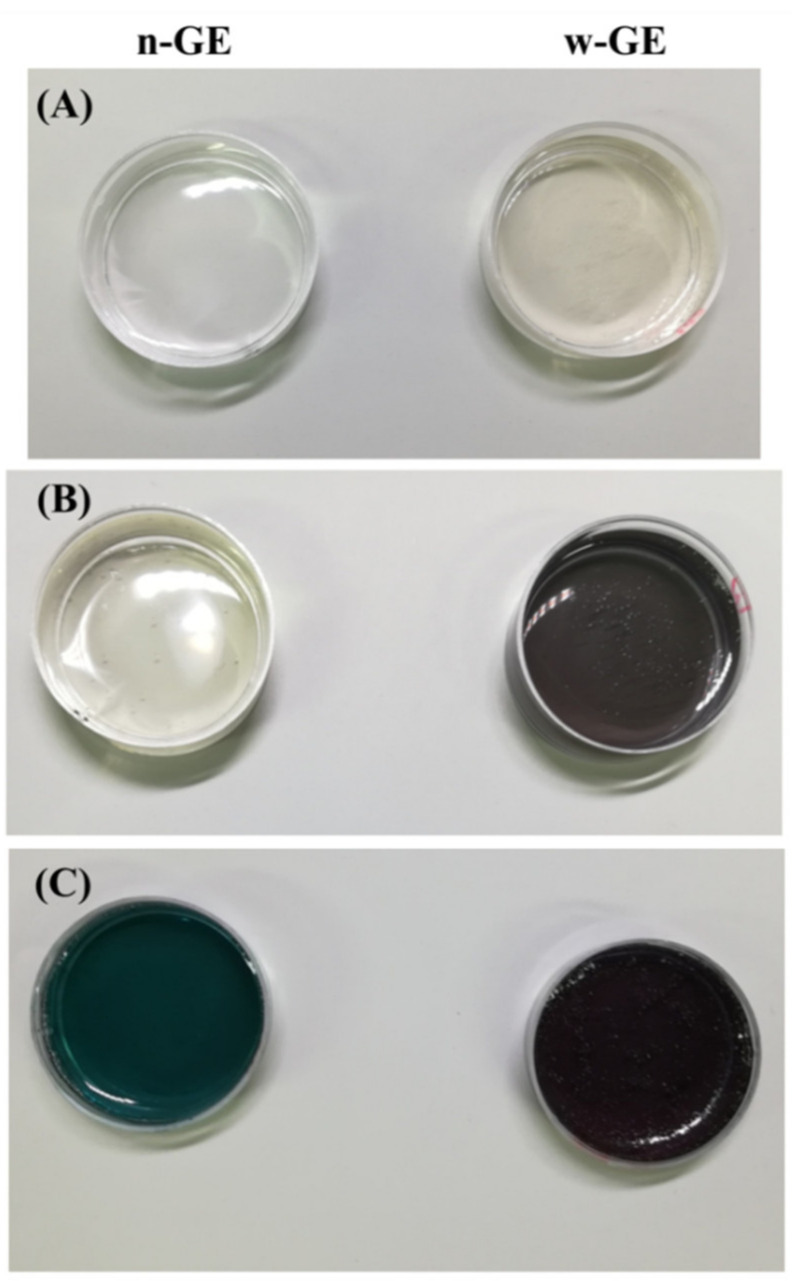
Appearance of CH/genipin solutions immediately after preparation (**A**), after 1 h (**B**), and after 6 h (**C**).

The genipin-induced gelations were accompanied by a characteristic blue pigmentation (Figure 2). The CH/w-GE solution was slightly yellowish immediately after preparation because of the browning of w-GE, while the CH/n-GE solution was colorless (Figure 2A). The CH/w-GE solution already formed a gel and turned dark purple 1 h after the preparation, while the CH/n-GE solution was still fluid and almost colorless (Figure 2B). The CH/n-GE solution also caused gelation and turned dark greenish-blue 6 h after preparation (Figure 2C).

### 3.2. Long-Term Monitoring of Gelation of CH/Genipin Solutions

**Figure 3:**
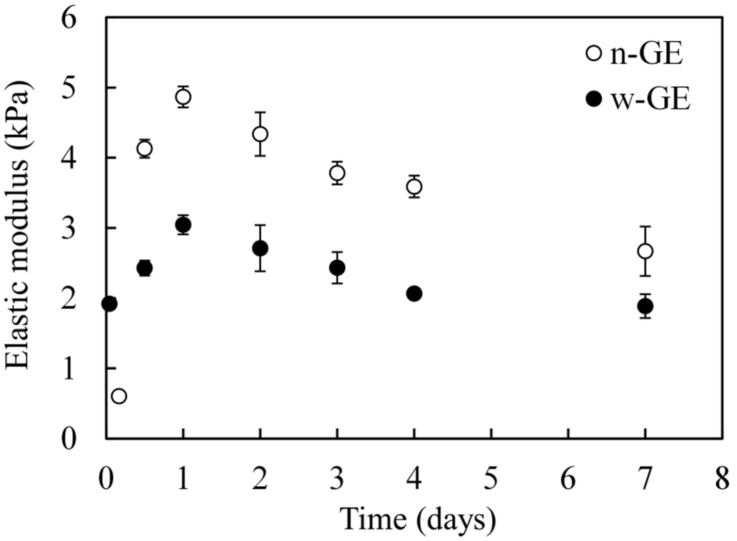
Time-dependent changes of elastic moduli of 0.8% CH/1 mM genipin gels. Data are expressed as mean ± SD (*n* = 5).

The mechanical tests for the CH/genipin gels were carried out to determine the time-dependent changes in elastic moduli of the gels over a period of 7 days (Figure 3), which is a reflection of crosslinking densities. The elastic modulus of the CH/w-GE gel increase time-dependently, reached its maximal value (3.05 kPa ± 0.14 kPa) in 1 day, and then began to decrease. Eventually, the elastic modulus leveled off at approximately 2 kPa from day 4 to day 7. The elastic modulus of the CH/n-GE was smaller than that of the CH/w-GE in the initial stage of the tests, but exceeded it at 12 h. It reached its maximal value (4.86 kPa ± 0.15 kPa) was also obtained in 1 day and was higher than that of the CH/w-GE gel. Subsequently, the elastic moduli decreased in a time-dependent manner and became 2.67 kPa ± 0.35 kPa at day 7.

### 3.3. Acute Cytotoxicity Tests of Genipin

**Figure 4:**
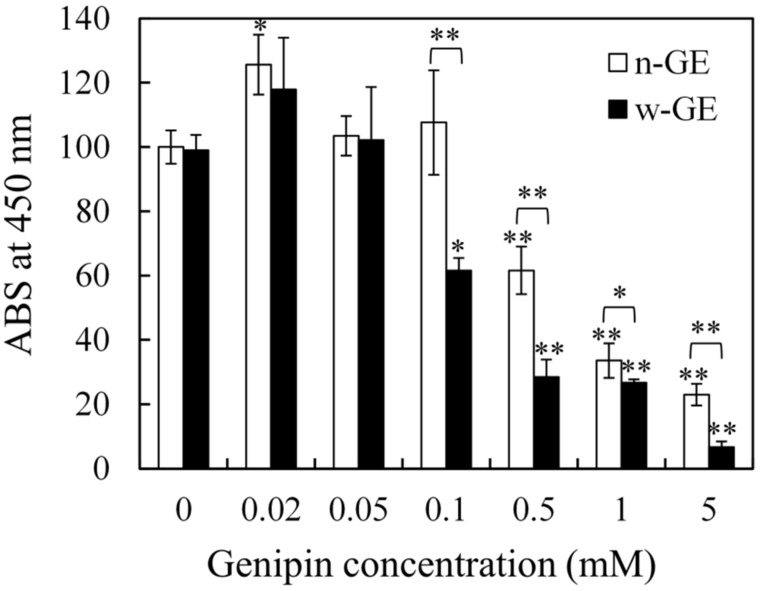
Results of acute cytotoxicity tests for w-GE and n-GE using a WST-8 assay. In this assay ABS at 450 nm reflects the numbers of viable V79 cells, which were in contact with genipin solutions for 1 h. Percent cell viability is the ratio of each ABS (genipin concentrations 0.02–5 mM) to that of the control (n-GE concentration = 0 mM). Statistical analyses were performed to evaluate statistical significances not only between various concentrations of genipins (0.02–5 mM) and the controls (without genipin) but also between w-GE and n-GE at similar concentrations. The results of the former statistical analyses are depicted over the bars. Those of the latter are depicted using brackets. *, *p* < 0.05; **, *p* < 0.01. Data are presented as mean ± SD (*n* = 3).

Figure 4 shows the results of the WST-8 assay for V79 cells, in which the concentrations of w-GE and n-GE were used in the range of 0−5 mM. The ABS from the cells treated with genipin increased concomitantly with the increase in genipin concentration from 0 to 0.02 mM (not statistically significant in w-GE). At higher concentrations of genipin, ABS began to decrease. The ABS at w-GE concentrations ≥0.1 mM and n-GE concentrations ≥0.5 mM were significantly lower than that of the controls (containing no genipin). The decrease in ABS from the cells treated with w-GE was greater than those treated with n-GE. As a result, the ABS in the w-GE experiments was significantly lower than that in the n-GE experiments at similar concentrations (0.1–5 mM). In these experiments, the cytotoxic level of w-GE at concentrations of 0.1 mM, 0.5 mM, and 1 mM were similar to those of n-GE at concentrations of 0.5 mM, 1 mM, and 5 mM, respectively. The microscopy images of cells damaged with w-GE and n-GE (data not shown) were similar to those observed in a previous study using n-GE and GA [17].

### 3.4. Cumulative Cytotoxicity Tests of Genipin

**Figure 5:**
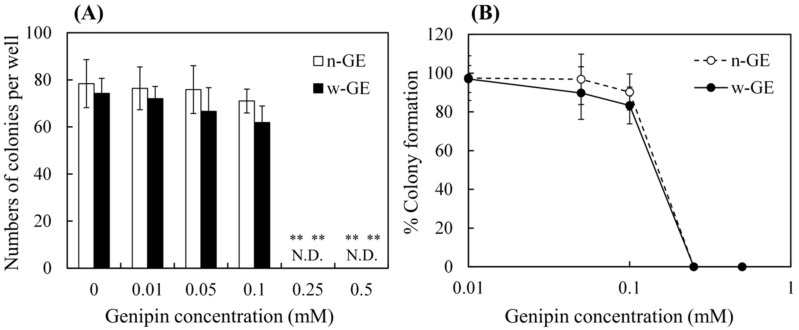
Results of cumulative cytotoxicity tests for w-GE and n-GE by a colony counting method. In these assays, colonies of V79 cells were counted after 7 days of culture. The bar graph (**A**) shows the number of colonies, in which statistical analyses were performed to evaluate statistical significance between various concentrations of genipins (0.02–5 mM) and the control (without genipin): **, *p* < 0.01. On the other hand, statistical analyses in the line graph (**B**) were performed to evaluate statistical significance between w-GE and n-GE at similar concentrations. There were no statistical differences between the two groups. Data are presented as mean ± SD (*n* = 8).

Figure 5 shows the results of cytotoxicity tests for w-GE and n-GE by the ISO 10993-5 standard method, which provides cumulative cytotoxicity for V79 cells over the period of 7 days. The cells survived at genipin concentrations 0–0.05 mM, but the numbers of colonies exhibited significant decreases when w-GE and n-GE concentrations reached 0.1 mM and 0.25 mM, respectively (Figure 5A). Colonies were no longer detected at a concentration ≥ 0.25 mM. The results of colony counting were converted to percent colony formations (Figure 5B). The lines of w-GE and n-GE almost overlapped; there were no statistical differences between the percent colony formations at the same concentrations. IC50 for w-GE and n-GE was determined to be 0.173 mM and 0.166 mM, respectively.

### 3.5. FTIR Spectra of Genipin and Crosslinked CH

**Figure 6:**
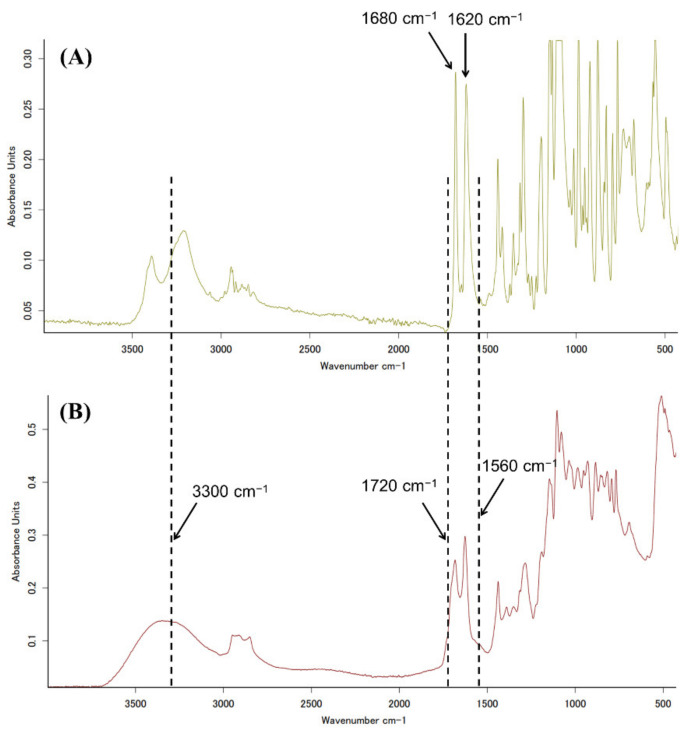
FTIR spectra of dried w-GE (**A**) and n-GE (**B**). Both the spectra were obtained with an ATR system.

Figure 6A,B show the FTIR spectra for n-GE and w-GE, respectively. The spectrum of w-GE was broad compared to that of n-GE. The major peaks at 1683 cm^−1^ and 1620 cm^−1^ for n-GE remained present in w-GE. However, shoulders of the peaks (approximately 1720 cm^−1^ and 1560 cm^−1^) appeared in w-GE. The two peaks at 3500–3000 cm^−1^ in n-GE turned into a broad single peak at approximately 3300 cm^−1^.

## 4. Discussion

In the present study, we experimentally determined the IC50 of w-GE (0.173 mM) and n-GE (0.166 mM) by the ISO 10993-5 standard method, which is established specifically for evaluating the cytotoxicity of medical devices. Genipin contained in biomaterials is typically used as a chemical cross linker for polymer substrates, not as a drug with pharmacological effects. Thus, the standard method is suitable for evaluating the potential cytotoxicity of genipin. To the best of our knowledge, the IC50 of w-GE and n-GE was determined by an international standard method for the first time. The IC50s obtained were similar to the IC50 for n-GE estimated by Sung et al. [17] by means of an unstandardized cytotoxicity tests for 3T3 fibroblasts using an MTT assay (≈0.44 mM) using 24-h cultivation and colony counting 10 days after cultivation (0.22–0.44 mM). This strengthens the reliability of our results. In the literature, the cytotoxicity of n-GE was estimated to be three orders of magnitude lower than that of GA. Thus, the identical IC50 of the two genipins is encouraging and makes us confident in the use of w-GE as a biologically safe and effective cross linker for in-situ forming gels [22].

IC50 is one of the most reliable and universal in vitro indices of cytotoxicity for chemicals, but, in this study, we took a cautious approach to evaluate cytotoxicity of w-GE by way of a custom-made acute cytotoxicity tests using a WST-8 assay. This course was decided based on the fact that the CH/w-GE solution completed its logarithmic phase of gelation by genipin-induced crosslinking at the 1 h time point after preparation (Figure 1), while the gelation of the CH/n-GE solution was much slower and still in a linear phase (Figure 1 and Figure 3). We employed a serum-free medium to avoid the consumption of genipins by reaction with serum proteins, allowing genipins to contact with cells directly. The acute cytotoxicity of w-GE was significantly higher than that of n-GE at concentrations 0.1–5 mM (Figure 4), reflecting the difference in crosslinking rates between these genipins. The pigmentation of the CH/genipin gels demonstrated that chemical reactions of n-GE were smaller than those of w-GE in the test period (1 h) (Figure 2), in which blue pigmentation occurred by the reaction between genipin and amino residues [25]. This difference in the rate of chemical reactions was also supported by the mechanical properties of the CH/genipin gels (Figure 3). The acute cytotoxicity of w-GE should be taken into consideration in its practical uses despite the fact that is it much quicker at crosslinking than n-GE. Furthermore, we should note that the ISO 10993-5 standard method cannot distinguish between acute and cumulative cytotoxicity of in-situ crosslinkers.

**Figure 7:**
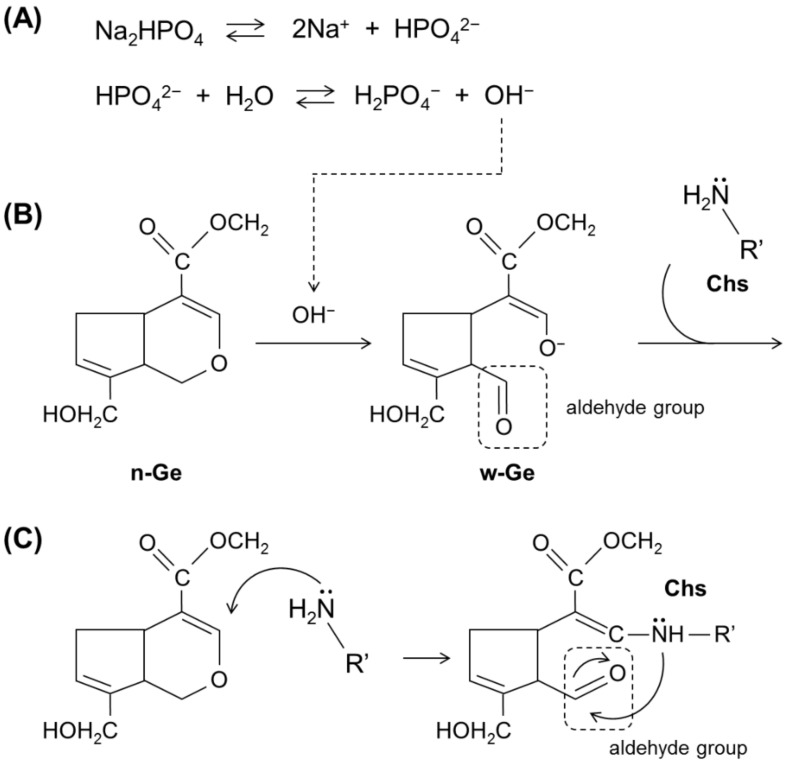
Presumed mechanism of initiation for genipin-induced crosslinking between biopolymers. (**A**) Release of hydroxyl ions from disodium hydrogen phosphate. (**B**) Aldehyde formation on genipin molecules due to the nucleophilic attack by hydroxyl ions. (**C**) Initiation of genipin-induced crosslinking, which is well understood [3,13,25] as a nucleophilic attack of free amine group to the C-3 carbon of genipin to form aldehyde group.

The acute cytotoxicity of w-GE can be explained by the formation of aldehyde groups. The shoulder at absorptions near 1720 cm^−1^ observed only in w-GE is assigned as a C=O stretch of aldehyde groups [14] (Figure 6B), which was also confirmed by ^13^C NMR measurements in our previous study [21]. The remaining two major peaks in the double bond regions (1683 cm^−1^ and 1620 cm^−1^, assigned as a C=O stretch and a C=C stretch of ester and cycloolefin of genipin) suggested that the ester group of the C-1 carbon and the double bond of the dihydropyran ring are not change by the preparation of w-GE. The other shoulder at absorptions near 1560 cm^−1^ in w-GE was attributed to the change of the chemical structure of the C-3 carbon. Figure 7B indicates a possible scheme for the formation of w-GE and subsequent reaction with free amine groups of biopolymers, based on the ring-opening reaction of genipin under alkaline conditions proposed by Mu et al. [15] We preliminary confirmed that w-GE was not prepared from n-GE by warming in pure water and sodium chloride solutions. Thus, hydroxyl ions from sodium dihydrogen phosphate (Figure 7A) are likely to make nucleophilic attack on n-GE (Figure 7B). The following crosslinking of biopolymers could occur by the reaction between carbonyl carbon of ester groups and free amine groups of biopolymers [13] or the dimerization of genipins by radical reactions [3], which are still controversial.

In contrast to the secondary reaction of genipin, it is commonly understood that the initiation of genipin-induced crosslinking occurs by nucleophilic attack of free amine group to the C-3 carbon of genipin to form aldehyde groups (Figure 7C) [3,13,25]. The slower gelation observed in n-GE (Figure 1 and Figure 3) suggests that this reaction occurs gradually over the period of 1 day. Assuming that the cytotoxicity of genipin is attributed to the chemical reactivity of aldehyde groups, the cumulative cytotoxicity of w-GE and n-GE must be equal because the cumulative molar mass of aldehyde groups depends on the initial concentration of genipins.

We explain the differences in acute and cumulative cytotoxicity of w-GE and n-GE based on the formation of aldehyde groups, but other complex genipin reactions may also contribute to the cytotoxicity of genipins. The modulus of the CH/genipin gels decreased as the incubation time increased (Figure 3). Similar results have been reported in the mechanical properties of mammalian tissues crosslinked with genipin [26]. These decreases in mechanical properties were explained by the lengthening of crosslinks by the polymerization of genipin [26]. It is conceivable that genipin molecules released from genipin-protein conjugates posed cytotoxic properties over the period of cell cultivation.

## 5. Conclusions

We determined the IC50 of w-GE (0.173 mM) and of n-GE (0.166 mM) by the ISO 10993-5 standard method, suggesting that cumulative cytotoxicity of w-GE is identical to that of n-GE. The IC50 (>0.1 mM) suggests that the genipins often exhibit non-toxicity when they are contained in medical devices. The acute cytotoxicity of w-GE was higher than that of n-GE, corresponding to much faster crosslinking properties for w-GE. Although the acute cytotoxicity of w-GE should be taken into consideration in its practical uses, it could be useful as an active cross linker for various in-situ forming gels.

## Data Availability

The data presented in this study are available on request from the corresponding author.
